# The Prevalence and Associated Factors of Job Burnout Among Medical Workers at COVID-19 Vaccination Sites: A Cross-Sectional Study

**DOI:** 10.1155/jonm/1280959

**Published:** 2025-04-09

**Authors:** Sijun Liu, Yinan Qian, Lili Gou, Lei Yuan, Lijun Lu, Mohammad Sulaiman Fadhi Al-shdifat

**Affiliations:** ^1^Department of Social Medicine and Health Education, School of Public Health, Nanjing Medical University, Nanjing, China; ^2^Department of Health Education, Jiangdu District Center for Disease Control and Prevention, Yangzhou, China; ^3^Department of Health Education, Nanjing Municipal Center for Disease Control and Prevention, Nanjing, China

**Keywords:** COVID-19, job burnout, medical workers, pandemic, vaccination

## Abstract

**Background:** During the pandemic period of the COVID-19, temporary centralized vaccination sites were set up in each administrative district in Nanjing to efficiently manage the vaccination campaign. Medical workers at COVID-19 vaccination sites are exposed to burnout syndrome due to repetitive and overload vaccination work. The purpose of our study was to investigate the prevalence of burnout among these medical workers and to explore its associated factors.

**Methods:** A cross-sectional study was conducted at COVID-19 vaccination sites in May 2021 in Nanjing, China. The online questionnaire included demographic, job and COVID-19-related characteristics, Chinese Maslach Burnout Inventory, and Social Support Rating Scale. The hierarchical multiple regression model was used to identify the risk factors for job burnout of medical workers.

**Results:** Of the 425 respondents, 189 had job burnout. The overall prevalence of burnout symptoms among medical workers at COVID-19 vaccination sites was 44.5% with a breakdown in severity as follows: 122 (28.7%) mild, 53 (12.5%) moderate, and 14 (3.3%) severe cases. Hierarchical multiple linear regression analysis indicated that education level, job titles, self-reported increased work intensity, self-assessment risk of contracting COVID-19 during work, and social support were significantly related to job burnout (*p* < 0.05), which explained 28.2% of the variance of job burnout score (*F* = 14.879, *p* < 0.01).

**Conclusion:** The burnout symptoms were relatively common among medical workers at COVID-19 vaccination sites. More attention should be paid to medical workers with master degree or higher, junior job titles, increased work intensity, high risk of contracting COVID-19 during work, and low level of social support. Interventions that aim to reduce workload and increase social support can be effective approaches to prevent job burnout among medical workers during controlled COVID-19 period.

## 1. Introduction

The COVID-19 pandemic, which emerged in 2019, has evolved into a public health crisis, continuing to pose significant challenges to global public health systems. Medical workers, in particular, have faced immense pressures due to high workload, elevated infection risks, resource shortages, and disruptions to work–life balance [1]. These stressors have led to mental health issues including chronic stress, anxiety, depression, and even job burnout [2, 3].

The term “job burnout” was first introduced by Freudenberger and is used to refer to a state of exhaustion and fatigue caused by constant emotional input of individuals in the service industry [4]. Job burnout was divided into three dimensions: emotional exhaustion (EE), depersonalization (DP), and decreased personal accomplishment (DPA) [5]. EE is characterized by lack of energy, enthusiasm, and fatigue. DP refers to an indifferent and detached attitude toward those individuals under one's care. DPA is marked by feelings of performing work tasks inadequately [6].

In recent years, it has been demonstrated that medical workers were more likely to experience job burnout than other professionals due to the high requirements and stressful risk of work [7, 8]. Job burnout among medical workers would lead to a negative impact on the physical and mental health of the individual, affect daily diagnosis and treatment, and even affect social stability [9–12]. A study of physicians in the UK and Ireland pointed out that job burnout was becoming an increasingly common phenomenon within the medical profession [13]. A systematic review of 182 studies involving 109,628 physicians in 45 countries demonstrated the overall burnout prevalence ranged from 0% to 80.5% [14]. During the initial COVID-19 pandemic, a survey conducted among medical workers from 69 countries all around the world reported a job burnout prevalence of 51% [15]. Meanwhile, a cross-sectional study in 133 cities in China showed that 36.5% of front-line medical staffs were suffering from job burnout [16].

Previous studies widely confirmed that job burnout is associated with social support [17], which refers to the material and spiritual support that an individual can obtain from the outside when facing stressful events, acting as an external resource in the process of reducing burnout [18]. Social support has a beneficial buffering effect and perform a meditating role between individuals' job stress and burnout [17, 19]. In addition, several demographic and job characteristics were suggested to be influencing factors in job burnout among medical workers based on previous studies, such as gender, marital status, health condition, monthly income, and working hour [6, 20–22].

According to China's National Bureau of Statistics, there were 102,314 confirmed cases of COVID-19 and 4636 deaths in 2021, China. Vaccination is one of the most effective and economical health measures to prevent and control COVID-19 [23]. It has been reported that a total of 2835.33 million doses of SARS-CoV-2 vaccines were administered nationwide in 2021 [24]. Besides traditional vaccination sites, temporary centralized vaccination sites were set up in each administrative district in China to cope with the high demand for vaccination and the limited capacity of traditional vaccination sites. These vaccination sites usually have the characteristics of high flexibility and rapid deployment, which can quickly expand the coverage of vaccination, especially during the outbreak of the epidemic, and effectively improve the efficiency and convenience of vaccination.

Given the limited original investigation during the prolonged public health crisis of COVID-19, we aimed to explore the prevalence and associated factors of job burnout among medical workers at temporary vaccination sites in Nanjing, China. We proposed the hypotheses that job burnout are associated with demographic, job, COVID-19-related characteristics, and social support.

## 2. Materials and Methods

### 2.1. Study Design

A cross-sectional study was conducted among medical workers at COVID-19 centralized vaccination sites in Nanjing from 13 to 26 May, 2021. The internet-based questionnaire was uploaded to the online professional survey platform of SO-JUMP in this study. In addition, there were guidelines to inform medical workers that their participation would be voluntary. Each account could only submit one questionnaire to avoid the possibility of repeated participation and each question was designed to be mandatory to avoid missing data. This study was approved by the Ethics Committee of Nanjing Medical University with the approval number FWA00001501. All subjects signed informed consent forms.

### 2.2. Study Participants

The study employed a convenience sampling technique to select participants in Nanjing. First, Xuanwu, Jianye, Pukou, and Jiangbei districts were selected from 12 districts in Nanjing. Second, all medical workers were selected as participants in our study from one of temporary vaccination sites in the abovementioned districts.

Participants were included in our study based on the following criteria: (1) taking part in the vaccination related work against Nanjing COVID-19 epidemic at the centralized sites, (2) being clinicians, nurses, medical technicians, and administrative staffs from community hospitals, and (3) consenting to participate in this survey.

The sample size was estimated using PASS15 based on the study design, expected statistical power, and estimated prevalence of job burnout. We assume that a 50% prevalence of burnout would be observed, the permissible absolute error was 5% and that *α* = 0.05 (two sided). A minimum sample size of 402 was required. Out of 434 participants selected, a total of 425 participants (48 male and 377 female) aged 21–58 years completed the survey with a response rate of 97.93%.

### 2.3. Measurements

The questionnaire was divided into four sections: sociodemographic characteristics, clinical information, the Chinese Maslach Burnout Inventory (CMBI), and the Social Support Rating Scale.

The first section covered the sociodemographic characteristics, including information concerning age (years), gender (“male” or “female”), marital status, and education level. Marital status was defined as married or unmarried (“single”, “divorced,” and “widowed”). Education level was recorded as “bachelor degree or below” and “master degree and above.”

The second section was clinical variables including job characteristics and COVID-19-related characteristics. The job characteristics included job tenure (years), working hours per day at centralized sits (“6-7,” “8-9,” and “≥ 10”), job title (“No title,” “Junior title,” “Middle title,” and “Senior title”), monthly income (“< 5000 RMB,” “5000–10000 RMB,” and “> 10,000 RMB”) and self-reported of increased work intensity after working in the centralized vaccination site (“Yes” or “No”). The COVID-19-related characteristics included self-assessment risk of contracting COVID-19 during work (“Low,” “Average,” and “High”), vaccination of COVID-19 (“Yes” or “No”).

The third section was the CMBI [25], which was developed from the Maslach Burnout Inventory and shown to adapt to the background of Chinese culture. The structure of the CMBI was also validated in three different occupational samples: teachers, police officers, and healthcare workers. The scale contains 15 items with three dimensions: EE (five items), DP (five items), and DPA (five items). Each item was measured by a seven-point Likert scale. Higher scores reflect greater job burnout. The severity of job burnout was classified into four levels—none, slight, moderate and severe according to the number of scores meeting or exceeding the cutoff values (i.e., EE ≥ 25, DP ≥ 11 and DPA ≥ 16). None job burnout was defined as no score met the cutoff values. Slight, moderate, and severe job burnout were defined as one, two, and three number of three scores met or exceeded cutoff values, respectively [26]. In this study, the Cronbach's *α* coefficient for CMBI was 0.824. A comprehensive evaluation of the questionnaire was performed using EFA, which revealed that it possessed a high degree of construct validity, as evidence by Kaiser–Meyer–Olkin (KMO) value of 0.848, and a high significant result (*p* < 0.001) in Bartlett's test of sphericity.

The forth section was the Social Support Rating Scale [27], which reflected staff member's perceived support from colleagues, supervisors, spouses, family members, and friends. The scale contains 12 items, ranked on a five-point Likert scale from 0 (never) to 4 (very much). Higher score reflects higher level of social support. The scale of the Chinese version showed good reliability and validity. Cronbach's *α* coefficient for the scale in this study was 0.920, which indicated acceptable internal consistency. A comprehensive evaluation of the questionnaire was performed using EFA, which revealed that it possessed a high degree of construct validity, as evidence by the KMO value of 0.830, and a high significant result (*p* < 0.001) in Bartlett's test of sphericity.

### 2.4. Statistical Analyses

Continuous variables were described using the mean and standard deviation. Differences among groups were compared by utilizing the *t*-test and one-way ANOVA. Categorical variables were described using frequency and percentage. Hierarchical multiple regression was performed to select variables that were responsible for the largest proportion of the explained variance. Only variables with *p* < 0.1 in univariate analysis were kept in the hierarchical multiple regression. All analyses were conducted using the Statistical Package for Social Sciences for Windows Version 26.0 (SPSS, Chicago, IL). *p* < 0.05 was considered statistically significant.

## 3. Results and Tables

### 3.1. Results

#### 3.1.1. Characteristics of Participants

A total of 425 participants completed the questionnaires and were enrolled in the final analyses. The average age and job tenure of the participants were 32.71 ± 7.53 and 11.11 ± 7.89 years, respectively. Most participants were females (88.7%) and married (72.7%). About 41.6% of them had middle title and senior title. Detailed demographic, job, and COVID-19-related characteristics are shown in Table 1.

#### 3.1.2. Job Burnout Among Medical Workers at Centralized Vaccination Sites

Among the 425 medical workers, the mean score of job burnout was 37.29 ± 12.26 and those of EE, DP, and DPA were 18.02 ± 7.88, 7.20 ± 3.52, and 12.08 ± 5.31, respectively. Overall, the results indicated severe EE rate of 22.1%, severe DP rate of 14.8%, and severe DPA rate of 26.6%. Moreover, the prevalence rates of slight, moderate, and severe burnout were 28.7%,12.5%, and 3.3%, respectively (Table 2).

#### 3.1.3. Univariate Analysis of Job Burnout Among Medical Workers

The results of association between demographic, job and COVID-19-related characteristics, and burnout among medical workers are shown in Table 1. There were significant differences in CMBI scores among different education level and job title groups for job burnout (*t* = −2.965, *p*=0.003, and *F* = 6.389, *p* < 0.001). Increasing work intensity was positively associated with job burnout (*t* = 6.448, *p* < 0.001). The “married” participants reported higher burnout score compared to those who were “unmarried” (*t* = −3.077, *p*=0.002). Participants who had increased work intensity reported higher scores compared with those who did not (*t* = −6.258, *p* < 0.001). Medical workers who assessed that they were more likely to be infected with COVID-19 during work had higher burnout scores (*F* = 11.544, *p* < 0.001).

#### 3.1.4. Hierarchical Multiple Regression Analysis Among Medical Workers

The results of hierarchical multiple linear regression analysis of the factors influencing job burnout scores were displayed in Table 3. For job burnout, only education level, job title, increased work intensity, self-assessment risk of contracting COVID-19 during work, and social support showed significant results in the final model with an adjusted *R*^2^ value of 0.282. Master degree or higher, junior job titles, increased work intensity, self-assessment high risk of contracting COVID-19 during work, and low level of social support predicted a high level of job burnout (*F* = 14.879, *p* < 0.001). The effects of different variables on the variance in job burnout score were displayed in the multiple linear regression model. Demographic-, job-, COVID-19-, and social support-related characteristics accounted for 4.1%, 8.7%, 2.2%, and 13.2% of the variance in job burnout, respectively.

## 4. Discussion and Conclusions

### 4.1. Discussion

The findings of this study reveal a high prevalence of burnout (44.5%) among medical workers at centralized COVID-19 vaccination sites in Nanjing, China. This rate is consistent with previous studies conducted in China during the COVID-19 pandemic, which reported burnout rates ranging from 37.39% to 73.98% among healthcare workers [28, 29]. The variation in burnout rates across studies may be attributed to differences in study settings, timing, and the intensity of the pandemic during data collection. For instance, Fu et al. reported a burnout rate of 37.39% among medical staff in Wuhan 1 year after the pandemic began [30], while a study conducted during the initial outbreak in February 2020 found a much higher burnout rate of 73.98% [31]. These comparisons suggest that burnout among medical workers in China is a persistent and serious issue, exacerbated by the prolonged nature of the pandemic and the high-stakes environment of vaccination efforts. The high prevalence of burnout underscores the need for targeted interventions to mitigate its impact on healthcare workers.

Our study found that medical workers with a master's degree or higher were more likely to experience burnout compared with those with a bachelor's degree or lower. This finding aligns with existing theories on occupational stress, which suggest that individuals with higher educational qualifications often face greater job responsibilities and expectations, leading to increased mental stress [32]. For example, medical professionals with advanced degrees are typically tasked with solving complex clinical problems and making critical decisions, which can contribute to emotional exhaustion and depersonalization, key dimensions of burnout [33]. This finding is consistent with previous research indicating that higher educational attainment is associated with increased job-related stress and burnout among healthcare professionals [34]. Therefore, interventions aimed at reducing burnout should consider the specific needs of highly educated medical workers, such as providing access to mental health resources and stress management programs.

Increased work intensity was significantly associated with higher levels of burnout among medical workers in this study. This finding is consistent with previous research conducted during the COVID-19 pandemic, which identified workload as a major predictor of burnout among healthcare professionals. For example, Liu et al. found that higher work intensity during the COVID-19 outbreak in China was strongly correlated with increased burnout levels [31]. Similarly, Amir et al. reported that increased workload was a significant predictor of burnout among nurses in Uganda during the pandemic [35]. According to the Job Demands–Resources (JD-R) model, excessive job demands, such as high work intensity, can deplete an individual's emotional and physical resources, leading to burnout [36]. In the context of centralized vaccination sites, the repetitive and high-pressure nature of the work may have contributed to a lack of job satisfaction and emotional exhaustion among medical workers. To address this issue, healthcare organizations should consider implementing measures to reduce workload, such as optimizing staffing levels and providing adequate breaks during shifts.

The study findings indicated that gender and marital status did not significantly influence job burnout among medical workers, which was not consistent with the reports by previous study [37]. However, our findings align with recent studies that suggest the extraordinary stressors of the pandemic may have equalized the impact on job burnout across different gender and marital status [38]. In our analysis, we noted that daily working hours did not significantly influence job burnout among healthcare workers at COVID-19 vaccination sites. The uniformity in the intensity and stress of the work environment during the pandemic may have overshadowed the impact of work duration on burnout [39]. The critical nature of the vaccination effort and the high-stakes environment likely imposed a baseline level of stress that rendered typical variations in work hours less influential.

Social support emerged as a critical protective factor against burnout in this study. Social support refers to the assistance and protection provided by others, whether in a formal setting (such as supervisors) or in an informal capacity (such as family members or coworkers) [40]. Yang et al. observed that self-reported social support was negatively associated with burnout syndrome in medical workers of southwest China [41]. A survey in nursing home workers during the COVID-19 pandemic in Spain found that perceived social support had a protective effect on burnout [42]. There is evidence that social support from occupational environment, such as coworker and supervisor support can help reduce burnout, thus increase the quality of health care [43]. This relationship can be explained by the fact that social support is an important external work resource that can prevent and reduce stress, alleviating occupational burnout. Considering the protective effect of social support on health [44], job burnout can be prevented or alleviated by actively providing appropriate social support for medical workers in varied ways. In addition, improving medical workers' perception and utilization of social support is an important measure to prevent and reverse burnout [45].

However, this study has the following limitations. First, this study was a cross-sectional design. Job burnout and associated factors were measured simultaneously. Therefore, it was impossible to draw causal relationships between them. Second, the participant sample was from centralized vaccination sites in Nanjing, China, which limited the applicability of the results to relevant populations in other cities. Third, the proportion of female medical workers is relatively high. The urgency of the pandemic and the intense workload faced by these workers posed significant challenges in implementing a stratified sampling approach, which requires that future studies should focus on the proportion of medical staff. Finally, we did not collect the information about the type of professionals which may contribute to job burnout. Further studies are required to elucidate the relationship between type of professionals and burnout.

### 4.2. Conclusions

In summary, job burnout among medical workers was common at centralized COVID-19 vaccination sites in Nanjing, China, during the controlled COVID-19 period. The result of our study showed that high level of job burnout was associated with high educational level, junior job title, increased work intensity, high risk of contracting COVID-19 during work and low level of social support. Therefore, it is necessary to provide social support at various levels and pay high attention to the physical and mental health of medical workers. Efforts also should be made to rationally arrange the workload of medical workers, thereby releasing job burnout and promote the quality of vaccination.

## Figures and Tables

**Table 1:** Participants' characteristics and association with job burnout among medical workers.

Variables	Categories	*N* (%)	M ± SD	*t*	*F*	*p*
*Demographic characteristics*						
Age (year)	< 30	165 (38.8)	35.88 ± 12.17		1.720	0.162
	30–39	187 (44.0)	38.76 ± 12.58			
	40–49	57 (13.4)	37.00 ± 11.51			
	≥ 50	16 (3.8)	35.81 ± 11.11			

Gender	Male	48 (11.3)	40.08 ± 12.56	1.677		0.094
	Female	377 (88.7)	36.94 ± 12.19			

Marital status	Unmarried^∗^	116 (27.3)	34.34 ± 11.45	−3.077		0.002
	Married	309 (72.7)	38.40 ± 12.39			

Education level	Bachelor degree or lower	403 (94.8)	36.89 ± 12.14	−2.965		0.003
	Master degree or higher	22 (5.2)	44.77 ± 12.38			

*Job characteristics*						
Job tenure (year)	≤ 10	241 (56.7)	37.14 ± 12.73	−0.294		0.769
	> 10	184 (43.3)	37.49 ± 11.64			

Hours/day	6–7	57 (13.4)	34.88 ± 10.71		6.448	0.002
	8–9	237 (55.8)	36.16 ± 12.64			
	≥ 10	131 (30.8)	40.39 ± 11.67			

Increased work intensity	Yes	372 (87.5)	38.64 ± 11.84	−6.258		< 0.001
	No	53 (12.5)	27.85 ± 11.00			

Job title	No	45 (10.6)	29.91 ± 9.21		6.389	< 0.001
	Junior	203 (47.8)	38.17 ± 12.48			
	Middle	142 (33.4)	37.95 ± 12.15			
	Senior	35 (8.2)	39.03 ± 12.08			

Monthly income (RMB)	< 5000	155 (36.5)	38.81 ± 12.76		1.934	0.146
	5000–10,000	254 (59.7)	36.50 ± 11.99			
	> 10,000	16 (3.8)	35.31 ± 10.73			

*COVID-19-related characteristics*						
Self-assessment risk of contracting COVID-19 in the course of work	Low	192 (45.2)	34.64 ± 11.81		11.544	< 0.001
	Average	136 (32.0)	37.89 ± 11.98			
	High	97 (22.8)	41.72 ± 12.25			

Vaccination of COVID-19	Yes	391 (92.0)	37.20 ± 12.23	−0.525		0.600
	No	34 (8.0)	38.35 ± 12.22			

^∗^Unmarried includes single, divorced, and widowed.

**Table 2:** Prevalence of burnout at different levels among medical workers.

Groups	*N*	%
No burnout	236	55.5
Slight burnout	122	28.7
Moderate burnout	53	12.5
Severe burnout	14	3.3

**Table 3:** Hierarchical multiple linear regression of job burnout among medical workers.

Variables (reference group)		Model 1	Model 2	Model 3	Model 4
*Demographic characteristics*					
Gender (male)	Female	−0.093	−0.091^∗^	−0.093^∗^	−0.056
Marital status (unmarried)	Married	0.141^∗∗^	0.059	0.054	0.056
Education level (bachelor degree or lower)	Master degree or higher	0.137^∗∗^	0.132^∗∗^	0.128^∗∗^	0.115^∗∗^

*Job characteristics*					
Job title (no)	Junior	—	0.233^∗∗^	0.228^∗∗^	0.173^∗^
	Middle	—	0.186^∗^	0.176^∗^	0.146
	Senior	—	0.092	0.096	0.070

Hours/day (6–7)	8-9	—	−0.027	−0.029	−0.046
	≥ 10	—	0.075	0.053	0.016

Increased work intensity (no)	Yes	—	0.236^∗∗^	0.215^∗∗^	0.206^∗∗^

*COVID-19-related characteristics*					
Self-assessment risk of contracting COVID-19 during work (low)	Average	—	—	0.108^∗^	0.072
	High	—	—	0.170^∗∗^	0.123^∗∗^

*Social support*	—	—	—	—	−0.370^∗∗^
*R* ^2^		0.048	0.146	0.172	0.302
Adjusted *R*^2^		0.041	0.128	0.150	0.282
*F*		7.071^∗∗^	7.894^∗∗^	7.786^∗∗^	14.879^∗∗^

*Note:* In Model 1, demographic characteristics were added. In Model 2, job characteristics were added. In Model 3, COVID-19-related characteristics were added. In Model 4, social support was added.

^∗^ *p* < 0.05.

^∗∗^ *p* < 0.01.

## Data Availability

The data used to support the findings of this study are available from the corresponding author upon reasonable request.
